# Epicardial fat density, coronary artery disease and inflammation in people living with HIV

**DOI:** 10.1097/MD.0000000000032980

**Published:** 2023-03-03

**Authors:** Manel Sadouni, Marie Duquet-Armand, Mohamed Ghaiss Alkeddeh, Mohamed El-Far, Etienne Larouche-Anctil, Cécile Tremblay, Jean-Guy Baril, Benoit Trottier, Carl Chartrand-Lefebvre, Madeleine Durand

**Affiliations:** a Centre hospitalier de l’Université de Montréal (CHUM) Research Center, Montreal, Canada; b Université de Montréal, Montreal, Canada; c Radiology, CHUM, Montreal, Canada; d Microbiology, CHUM, Montreal, Canada; e Medical Clinic Quartier Latin, Montreal, Canada; f Internal Medicine, CHUM, Montreal, Canada.

**Keywords:** density, epicardial fat, HIV, inflammation, plaque, quality

## Abstract

Studies have shown an increased risk of coronary artery disease (CAD) in the human immunodeficiency virus (HIV) population. Epicardial fat (EF) quality may be linked to this increased risk. In our study, we evaluated the associations between EF density, a qualitative characteristic of fat, and inflammatory markers, cardiovascular risk factors, HIV-related parameters, and CAD. Our study was cross-sectional, nested in the Canadian HIV and Aging Cohort Study, a large prospective cohort that includes participants living with HIV (PLHIV) and healthy controls. Participants underwent cardiac computed tomography angiography to measure volume and density of EF, coronary artery calcium score, coronary plaque, and low attenuation plaque volume. Association between EF density, cardiovascular risk factors, HIV parameters, and CAD were evaluated using adjusted regression analysis. A total of 177 PLHIV and 83 healthy controls were included in this study. EF density was similar between the two groups (−77.4 ± 5.6 HU for PLHIV and −77.0 ± 5.6 HU for uninfected controls, *P* = .162). Multivariable models showed positive association between EF density and coronary calcium score (odds ratio, 1.07, *P* = .023). Among the soluble biomarkers measured in our study, adjusted analyses showed that IL2Rα, tumor necrosis factor alpha and luteizing hormone were significantly associated with EF density. Our study showed that an increase in EF density was associated with a higher coronary calcium score and with inflammatory markers in a population that includes PLHIV.

## 1. Introduction

The introduction of highly active antiretroviral therapy in the mid-90s has led to a decrease in the mortality related to human immunodeficiency virus (HIV) infection accompanied by an increase in life expectancy.^[1,2]^ Individuals living with HIV have a higher risk to develop age-related diseases such as coronary artery disease (CAD) and several cohort studies have shown an increased risk of CAD in people living with HIV compared with the general population.^[3–5]^ The pathophysiological processes underlying this increased risk are not well understood but may be linked to metabolic changes such as adipose tissue disturbances or inflammation.^[6,7]^

Epicardial fat (EF), which is the visceral fat of the heart, can be considered as an active organ capable of secreting inflammatory cytokines that may have local and systemic effects on cardiovascular disease (CVD).^[8,9]^ Several studies have shown that the amount of EF is increased in participants living with HIV (PLHIV), and that it is associated with a greater risk of CAD.^[10–12]^ On the other hand, only a limited number of studies have explored how EF quality, beyond its quantity, can be related to inflammation and cardiovascular health among PLHIV.^[13–15]^

Fat quality can be measured using fat computed tomography (CT) density (or CT attenuation, in Hounsfield units [HU]). This qualitative characteristic of fat can vary due to alterations in lipid content, tissue vascularity, and inflammation.^[13–17]^ Studies performed in the general population have shown that changes in fat density may impact CAD risk independently of fat quantity.^[13,18,19]^

In this study, we aimed to measure the associations between EF CT-measured density in both PLHIV and uninfected controls, and levels of inflammatory markers, cardiovascular risk factors, HIV-related parameters, and coronary artery plaque burden.

## 2. Methods

### 2.1. Study design and study population

We conducted a cross-sectional study nested within the Canadian HIV and Aging Cohort Study (CHACS), an ongoing multicenter prospective cohort following participants with and without HIV, with the aim of identifying the determinants of premature aging in people with HIV. Inclusion and exclusion criteria were previously reported.^[20]^

We report results from the CHACS cardiovascular imaging sub-study, in which consecutive CHACS participants, aged 40 years or older, with a 10-year Framingham risk score between 5% and 20%, without symptoms or history of CAD, with a preserved renal function and without allergy to contrast media were invited to undergo non-contrast cardiac CT and coronary CT angiography. This study was approved by the Institutional Review Board of the Medical Center of the University of Montreal and participating centers. All participants gave written informed consent.

### 2.2. Study procedures

Non-contrast cardiac CT and coronary CT angiography were performed using a 256-slice CT scanner (Brilliance iCT; Philips Healthcare, Best, The Netherlands). Prior to CT, participants were given 50 to 75 mg of metoprolol orally if heart rate was >60 beats/min, and 0.4 mg of nitroglycerin sublingually unless contraindicated.

Non-contrast cardiac CT was performed for coronary calcium scoring and EF evaluation using the following parameters: slice thickness 2.5 mm, matrix 512 × 512, field-of-view 250 mm, scan voltage 120 kV and prospective electrocardiographic-gating. CT angiography was performed for coronary plaque analysis using 370 mg/mL of iopamidol (Bracco Imaging, Milan, Italy) at a flow rate of 5 mL/s. Images were reconstructed using a hybrid iterative reconstruction algorithm (Philips iDose; Philips Healthcare, level 3).

### 2.3. Exposure of interest: EF density

EF is the fat deposit located between the visceral pericardium and the myocardium. EF density decreases with fat gain and increases with fat loss.^[16,21]^ In addition, EF density increases with inflammatory cell infiltration and metabolic dysfunction.^[14,16,22]^ Therefore, the higher the density, the more metabolically active the fat deposition might be.^[13,23]^

EF density and volume were both assessed using non-contrast CT images and a semi-automated software (Aquarius Intuition version 4.4.11; TeraRecon Headquarters, Forster City, CA). The pericardium was manually traced on axial slices from the pulmonary artery bifurcation to the apex of the heart. A CT density between −190 and −30 HU was used to select the EF and exclude any other tissue. Mean EF density and volume were calculated using the software based on the adipose tissue area, the number of slices, slice thickness, and intersection gaps, and reported as a continuous value, expressed in HU and cm^3^.

Two observers both blinded to HIV status and clinical data measured EF volume and density using the semi-automated software. Inter-observer and intra-observer agreement for EF volume and density measurement were highly reproducible (intraclass correlation coefficient for inter-observer agreement 0.75 for EF volume and 0.99 for density; Intra-observer agreement 0.97 for EF volume and 0.97 for density).^[24]^

### 2.4. Outcome of interest: coronary plaque

Coronary artery calcium (CAC) measurement was performed using the method by Agatston et al^[25]^ on non-contrast CT images. Coronary plaque analysis was performed using CT angiography images as previously described.^[26]^ Plaque volume was quantified in multiplanar reformat. Total plaque, calcified, non-calcified and mixed plaque volumes per participant were defined as the sum of volumes of all plaques, calcified, non-calcified and mixed plaques per participant. Low-attenuation plaque, a marker of plaque vulnerability, was defined as the sum of volumes of all portions of 30 HU or less of each plaque per participant.

Inter-observer and intra-observer agreement for plaque volume analysis was excellent as previously described.^[27]^

### 2.5. Metabolic and inflammatory biomarkers

Eighty-eight inflammatory, anti-inflammatory and metabolic markers were measured in the CHACS cohort. These biomarkers were measured in plasma collected from the study participants using regular ELISA assays and Meso Scale technology as previously described.^[28]^ In this study, we present results for 11 markers as these were the only one that were significantly associated to EF density in univariable analysis.

### 2.6. Statistical analysis

Continuous variables are reported as mean ± standard deviation or median [25th–75th interquartile range], as appropriate. Categorical variables are reported in frequency and percentages. Student *t* test or Mann–Whitney *U* test was used to compare normally and nonnormally distributed variables between participants with HIV and controls and chi-squared test was used to compare categorical variables.

The association of EF density with cardiovascular risk factors, as well as with HIV related parameters, was assessed using univariable and multivariable linear regression models, with and without inclusion of EF volume into the models. Multivariable model included all HIV-related parameters and cardiovascular risk factors risk factors significantly associated with EF density.

The association of EF density with coronary plaque (CAC, total, calcified, noncalcified and mixed plaque volume) was evaluated using zero-inflated Poisson regression, as previously described.^[10]^ The use of zero-inflated model is made necessary by the excess of 0 in the distribution of total coronary plaque volumes. Coronary plaque volume variables were natural-log transformed prior to statistical analysis. Multivariable models were adjusted for CVD risk factors.

In addition and as an exploratory analysis, the association of EF with inflammatory biomarkers was evaluated using univariable and multivariable linear regression models. Multivariable models included inflammatory biomarkers and CVD risk factors if they showed a univariable association with EF. As this analysis was hypothesis-generating, we did multiple tests and did not correct for multiple comparisons.

Effect modification by HIV of each association was assessed by inclusion of an interaction term to the fully adjusted models, and significance of the interaction term was assessed by a likelihood ratio test.

For patients with incomplete data, the mean or median value was used to impute the missing data. Values for the following number of participants were missing and imputed: Body mass index (BMI),^[5]^ smoking exposure,^[14]^ high density lipoprotein-cholesterol,^[4]^ low density lipoprotein-cholesterol,^[9]^ antiretroviral therapy (ART) exposure duration,^[7]^ non-nucleoside reverse transcriptase inhibitors exposure duration.^[7]^ A *P* value <.05 was considered statistically significant. Statistical analyses were performed using R (version 3.3.2; R Foundation for Statistical Computing, Vienna, Austria).

## 3. Results

### 3.1. Participant’s characteristics

Overall, 260 participants from the CHACS cohort were included in this study (177 (68.1%) PLHIV and 83 (31.9%) uninfected controls). Participant’s characteristics stratified by HIV status are listed in Table 1. PLHIV had a similar 10-year Framingham risk score (median of 10% [7–16] for PLHIV and 9% [7–15] for controls, *P* = .414). PLHIV were more likely to be male (92.0% for PLHIV vs 77.1% for controls, *P* = .002), to smoke more (median smoking exposure of 6 [0–24.4] pack/years for PLHIV vs 0 [0–8.4] for controls, *P* = .001) and had a lower cholesterol level (mean total cholesterol of 4.9 ± 1.1 for PLHIV vs 5.3 ± 1.0 for controls, *P* = .003). PLHIV had a lower BMI compared to uninfected controls (mean of 25.3 ± 4.1 kg/m^2^ for PLHIV vs 27.1 ± 4.3 kg/m^2^ for controls, *P* = .002) while waist circumference was similar between the 2 groups (94.7 ± 11.5 for PLHIV vs 95.3 ± 10.6 for controls, *P* = .675).

**Table 1:** Characteristics of study participants (N = 260).

Variables	HIV^+^ = 177	HIV^−^ = 83	*P* value
**CVD risk factors**			
Age (yr)	55.8 ± 7.0	56.5 ± 8.0	.470
Male sex, n (%)	163 (92%)	64 (77.1%)	**.002**
Race, n (%)			
Asian	3 (1.7%)	0 (0%)	.227
Black	17 (9.6%)	2 (2.4%)	
Caucasian	147 (83%)	77 (92.8%)	
Hispanic	9 (5.1%)	4 (4.8%)	
Diabetes, n (%)	17 (9.6%)	3 (3.6%)	.150
High blood pressure, n (%)	56 (31.6%)	19 (22.9%)	.192
Family history of premature CVD, n (%)	37 (20.9%)	18 (21.7%)	1
Smoking status, n (%)			
Current	53 (29.9%)	9 (10.8%)	**.001**
Ex	65 (36.7%)	34 (41.0%)	
Never	55 (31.1%)	39 (47.0%)	
Smoking exposure (pack/year)	6 [0–24.4]	0 [0–8.4]	**.001**
Total-Cholesterol (mmol/L)	4.9 ± 1.1	5.3 ± 1.0	**.003**
HDL-cholesterol (mmol/L)	1.2 ± 0.4	1.4 ± 0.4	**.001**
LDL-cholesterol (mmol/L)	2.9 ± 0.9	3.2 ± 0.9	**.003**
Statin use, n (%)	52 (29.4%)	15 (18.1%)	.073
BMI (kg/m^2^)	25.3 ± 4.1	27.1 ± 4.3	**.002**
BMI categories			
Underweight	7 (3.9%)	0 (0%)	**.006**
Normal	77 (43.5)	23 (27.7%)	
Overweight	72 (40.7)	41 (49.4%)	
Obese	21 (11.9)	19 (22.9%)	
Waist circumference (cm)	94.7 ± 11.5	95.3 ± 10.6	.675
10-year Framingham risk score (%)	10 [7–16]	9 [7–15]	.414
**HIV-specific variables**	**HIV^+^ = 177**		***P* value**
HIV infection duration (yr)	18.3 ± 7.7		
Participants exposed to ART, n (%)	166 (93.8%)		
ART exposure duration (yr)*	13.5 ± 6.5		
Participants exposed to PIs, n (%)	132 (74.6%)		
PIs exposure duration (yr)*	9.5 ± 5.1		
Participants exposed to NRTIs, n (%)	166 (93.8%)		
NRTIs exposure duration (yr)*	12.4 ± 6.2		
Participants exposed to NNRTIs, n (%)	112 (62.3%)		
NNRTIs exposure duration (yr)*	4.5 [2.0–7.5]		
Participants exposed to INSTIs, n (%)	80 (45.2%)		
IIs exposure duration (yr)*	1.8 [0.9–3.8]		
Detectable viral load,† n (%)	15 (8.5%)		
Viral load among detectable	1135.0 [71.5–24,909.5]		
Nadir CD4 cell count (cells/mm^3^)	200 [100–300]		
Current CD4 cell count (cells/mm^3^)	611.7 ± 304.0		
Current CD8 cell count (cells/mm^3^)	831.9 ± 417.2		
**Inflammatory markers (N = 123**)	**HIV^+^ = 76**	**HIV^−^ = 47**	***P* value**
IL2Ra	711.5 [542.0–966.6]	604.09 [470.23–812.01]	.080
IL7	24.8 [19.9–30.0]	19.4 [14.9–21.8]	**<.001**
Leptin	3510.8 [861.5–8770.2]	3348.7 [979.1–7992.1]	.904
Peptide C	2115 [1399–3017]	1757.5 [1220.6–3141.3]	.306
Insulin	17.3 [11.0–37.1]	13.4 [9.2–27.8]	.194
TNFα	1.4 [1.1–2.0]	1.2 [0.7–1.5]	**.005**
ENA78	4272 [2965–5854]	3667 [2387–5119]	.159
GROα	1644.5 [1257.1–1955.3]	1299.2 [943.3–1628.9]	**<.001**
FSH	3581.0 [1855.6–6043.3]	2393.1 [1604.0–4377.9]	**.046**
LH	2344.3 [1644.5–3062.4]	1761.3 [1431.8–2746.6]	.089
PP	171.6 [103.7–303.4]	200.6 [111.2–598.6]	.055

Normally distributed variables are expressed as mean ± standard deviation, non-normally distributed variables are expressed as median [Q1–Q3], categorical variables are expressed using proportion (percentage). *P* values are unadjusted. Bold numbers to identify statistical significance.

ART = antiretroviral therapy, BMI = body mass index, CVD = cardiovascular disease, ENA = epithelial-derived neutrophil-activating, FSH = follicular stimulating hormone, GRO = growth-regulated oncogene, HIV = human immunodeficiency virus, IL = interleukine, INSTI = integrase strand transfer inhibitors, LH = luteizing hormone, NNRTIs = non-nucleoside reverse transcriptase inhibitors, NRTIs = nucleoside reverse transcriptase inhibitors, PIs = protease inhibitors, TNF = tumor necrosis factor.

* In patients exposed to therapy.

† Defined as viral load > 40 copies/mL.

Subjects with HIV had a mean duration of HIV infection of 18.3 ± 7.7 years. More than 93% of PLHIV were exposed to ART with a mean duration of 13.5 ± 6.5 years. 74.6% were exposed to proteas inhibitors, 93.8% where exposed to nucleoside reverse transcriptase inhibitors, 62.3% were exposed to NNRTIs and 45.2% were exposed to integrase strand transfer inhibitors. Viral load was detectable in 8.5% of PLHIV and mean CD4 count was 611.7 ± 304.0 cells/mm^3^.

All levels of circulating inflammatory markers were similar between the 2 groups except for interleukine-7 (IL7), tumor necrosis factor alpha (TNFα), growth-regulated oncogene alpha and follicular stimulating hormone which were higher in PLHIV compared to controls (all *P* < .05).

PLHIV had a greater EF volume (113.4 ± 49.2 cm^2^) than uninfected controls (101.7 ± 43.7 cm^2^) (*P* = .046), whereas EF density was similar between the 2 groups (−77.4 ± 5.6 HU for PLHIV and −77.0 ± 5.6 HU for uninfected controls, *P* = .162). There was no significant difference in presence of coronary calcium (66.1% in PLHIV and 57.8% in controls, *P* = .326) and in CAC score between the 2 groups (14.5 [0–127.8] for PLHIV and 8.3 [0–68.0] for controls, *P* = .196). At coronary CT angiography, total plaque volume showed no significant difference between PLHIV and controls (81.0 [0–357.9] for PLHIV and 40.7 [0–172.4] for controls, *P* = .052), however, PLHIV had a higher prevalence and volume of the non-calcified and mixed plaques compared to uninfected controls (prevalence of non calcified plaques for PLHIV 21.1% vs 7.8% for controls, *P* = .018, prevalence of mixed plaques for PLHIV 48.7% vs 31.2% for controls, *P* = .017). All cardiac CT results are presented in Table 2.

**Table 2:** Cardiac computed tomography results (N = 260).

Non-contrast cardiac CT (N = 257)	HIV^+^ = 177	HIV^−^ = 83	*P* value
Presence of coronary calcium	117 (66.1%)	48 (57.8%)	.326
Coronary artery calcium score	14.5 [0–127.8]	8.3 [0–68.0]	.196
Epicardial fat volume (cm^2^)	113.8 ± 49.2	101.7 ± 43.7	**.046**
Epicardial fat density (HU)	−77.4 ± 5.6	−77.0 ± 5.6	.582
**Coronary CT angiography (N = 226**)	**HIV^+^ = 152**	**HIV^−^ = 77**	***P* value**
Plaque presence	106 (69.7%)	46 (59.7%)	.172
Total plaque volume (mm^3^)	81.0 [0–357.9]	40.7 [0–172.4]	.052
Calcified plaque presence	69 (45.5%)	38 (49.3%)	.670
Calcified plaque volume (mm^3^)	0 [0–79.5]	0 [0–95.7]	.795
Non calcified plaque presence	32 (21.1%)	6 (7.8%)	**.018**
Non calcified plaque volume (mm^3^)	0 [0–0]	0 [0–0]	**.014**
Mixed plaque presence	74 (48.7%)	24 (31.2%)	**.017**
Mixed plaque volume (mm^3^)	0 [0–136.9]	0 [0–51.9]	**.010**
Low attenuation plaque volume (mm^3^)	17.9 [0.0–97.7]	6.4 [0.0–47.4]	.056

Normally distributed variables are expressed as mean ± standard deviation, non-normally distributed variables are expressed as median [Q1–Q3], categorical variables are expressed using proportion (percentage). *P* values are unadjusted. Bold numbers to identify statistical significance.

CT = computed tomography, HIV = human immunodeficiency virus.

### 3.2. EF density and CVD risk factors

Univariable analysis showed that EF density was negatively associated with age, with fat density decreasing with advancing age (β = −0.13 per 1 year increase in age, *P* = .004), smoking exposure (β = −0.04 per each additional pack-years of exposure, *P* = .026), triglyceride (β = −0.80 per 1 mmol/L increase in triglyceride, *P* = .015), statin use (β = −2.25 for use vs non use, *P* = .004), BMI (β = −0.32 per 1 unit increase in BMI, *P* < .001) and EF volume (β = −0.09 for each increase in 1 cm^3^, *P* < .001) while a positive association was observed with male sex (β = 3.03, *P* = .003). In a multivariable regression model including all the traditional cardiovascular risk factors and EF volume, only male sex, diabetes and EF volume were independently associated with EF density (Table 3).

**Table 3:** Association of cardiovascular risk factors with epicardial fat density in all participants, N = 260 (175 HIV+, 83 HIV−).

	Univariable analysis		Multivariable analysis*		Multivariable analysis†	
	Beta‡ (95% CI)	*P* value	Beta‡ (95% CI)	*P* value	Beta‡ (95% CI)	*P* value
Age (per 1 year increase)	−0.13 (−0.23 to 0.04)	**.004**	−0.13 (−0.23 to 0.04)	**.006**	−0.03 (−0.10 to 0.03)	.294
Male sex	3.03 (1.02–5.05)	**.003**	2.60 (0.46–4.75)	**.017**	3.11 (1.64–4.58)	**<.001**
HIV status	−0.41 (−1.87 to 1.05)	.582	−1.04 (−2.55 to 0.47)	.174	0.16 (−0.88 to 1.21)	.756
Diabetes	−1.73(−4.28 to 0.82)	.182	0.68 (−1.93 to 3.29)	.608	2.34 (0.54–4.15)	**.011**
High blood pressure	0.10 (−1.61 to 1.40)	.894	1.17 (−0.35 to 2.70)	.131	0.80 (−0.35 to 2.70)	.135
Family history of premature CVD	−0.25 (−1.92 to 1.42)	.768	0.002 (−1.60 to 1.61)	.997	0.58 (−1.69 to 0.52)	.298
Smoking exposure (per 1 pack/year increase)	−0.04 (−0.08 to 0.01)	**.026**	−0.03 (−0.07 to 0.002)	.064	0.01 (−0.02 to 0.03)	.671
HDL-cholesterol (per 1 mmol/L increase)	0.17 (−1.68 to 2.02)	.857	0.32 (−1.64 to 2.27)	.748	−0.02 (−1.36 to 1.32)	.976
LDL-cholesterol (per 1 mmol/L increase)	0.06 (−0.69 to 0.82)	.867	−0.05 (−0.82 to 0.72)	.899	−0.21 (−0.74 to 0.32)	.433
Triglycerides (per 1 mmol/L increase)	−0.80 (−1.46 to 0.15)	**.015**	−0.42 (−1.09 to 0.26)	.223	−0.10 (−0.57 to 0.36)	.669
Statin use	−2.25 (−3.78 to 0.71)	**.004**	−1.46 (−3.05 to 0.13)	.071	−0.48 (−1.58 to 0.61)	.382
BMI (per 1 kg/m^2^ increase)	−0.32 (−0.48 to 0.16)	**<.001**	−0.33 (−0.49 to 0.16)	**<.001**	0.11 (0.02–0.24)	.081
Epicardial fat volume (per 1 cm^3^ increase)	−0.09 (−0.10 to 0.08)	**<.001**	–	–	−0.09 (−0.10 to 0.08)	**<.001**

Bold numbers to identify statistical significance.

BMI = body mass index, CVD = cardiovascular disease, HIV = human immunodeficiency virus.

* Multivariable analyses included all cardiovascular risk factors (age, male sex, HIV status, diabetes, high blood pressure, family history of premature CVD, smoking exposure, HDL-cholesterol, LDL-cholesterol, triglycerides, statin use and BMI).

† Multivariable analyses included all cardiovascular risk factors (age, male sex, HIV status, diabetes, high blood pressure, family history of premature CVD, smoking exposure, HDL-cholesterol, LDL-cholesterol, triglycerides, statin use and BMI) and epicardial fat volume.

‡ Beta is interpreted as the mean increase in epicardial fat attenuation (in HU) predicted by the model per each one unit change in the explanatory variable. A negative beta coefficient indicates a decrease.

### 3.3. EF density and HIV-related parameters

In univariable models, EF density was only significantly associated with NNRTI exposure duration (β = −2.12, per 10 years increase of exposure, *P* = .031) while no association was observed with other ART classes, HIV duration, CD4 and CD8 count. In a multivariable regression model including all HIV-related parameters, CVD risk factors, and EF density, none of the HIV related parameters was significantly associated to EF density. Results are presented in Tables 4 and 5.

**Table 4:** Association of HIV specific variables with epicardial fat density in participants living with HIV, N = 177 (including overall antiretroviral therapy exposition duration).

	Univariable analysis		Multivariable analysis*		Multivariable analysis†	
	Beta‡ (95% CI)	*P* value	Beta‡ (95% CI)	*P* value	Beta‡ (95 CI)	*P* value
HIV infection duration (per 10 years increase)	−0.38 (−1.46 to 0.70)	.486	−0.23 (−1.56 to 1.11)	.738	0.08 (−0.83 to 1.01)	.858
ART exposition duration (per 10 years increase)	−0.73 (−2.00 to 0.50)	.245	−0.61 (−2.22 to 1.00)	.453	0.42 (−0.70 to 1.53)	.463
Nadir CD4 count (per 100 cells/mm^3^)	−0.08 (−0.68 to 0.51)	.783	0.12 (−0.53 to 0.76)	.719	0.19 (−0.26 to 0.63)	.409
Current CD4 count (per 100 cells/mm^3^)	−0.19 (−0.46 to 0.08)	.172	−0.16 (−0.48 to 0.15)	.311	−0.20 (−0.42 to 0.01)	.068
Current CD8 count (per 100 cells/mm^3^)	0.05 (−0.15 to 0.26)	.605	0.07 (−0.14 to 0.28)	.519	0.06 (−0.08 to 0.21)	.367
Detectable viral load (yes or no)§	3.15 (0.20–6.09)	.036	1.39 (−1.77 to 5.54)	.387	−0.04 (−2.14 to 2.23)	.969

ART = antiretroviral therapy, HIV = human immunodeficiency virus.

* Multivariable analyses included age, sex, cardiovascular risk factors if they showed an association with the outcome in the univariable model with a *P* value .1 or less and HIV related parameters including HIV infection duration, ART exposition duration, nadir CD4 count, current CD4 count, current CD8 and detectable viral load.

† Multivariable analyses included age, sex, EF volume, cardiovascular risk factors if they showed an association with the outcome in the univariable model with a *P* value .1 or less and HIV related parameters including HIV infection duration, ART exposition duration, nadir CD4 count, current CD4 count, current CD8 and detectable viral load.

‡ Beta is interpreted as the mean increase in epicardial fat attenuation (in HU) predicted by the model per each one unit change in the explanatory variable. A negative beta coefficient indicates a decrease.

§ Defined as viral load greater than 40 copies/mL (0: no detectable viral load, 1: detectable viral load).

**Table 5:** Association of epicardial fat density with HIV-specific variables in participants living with HIV, N = 177 (including antiretroviral therapy class exposition duration).

	Univariable analysis		Multivariable analysis*		Multivariable analysis†	
	Beta‡ (95% CI)	*P* value	Beta‡ (95% CI)	*P* value	Beta‡ (95% CI)	*P* value
HIV infection duration (per 10 years increase)	−0.38 (−1.46 to 0.70)	.486	−0.65 (−1.99 to 0.69)	.340	−0.04 (−0.98 to 0.90)	.933
PI exposition duration (per 10 years increase)	0.84 (−0.56 to 0.22)	.239	0.90 (−1.72 to 3.51)	.499	0.64 (−1.18 to 2.45)	.492
NRTI exposition duration (per 10 years increase)	−0.45 (−1.78 to 0.88)	.505	−0.24 (−3.01 to 2.52)	.863	0.16 (−1.75 to 2.08)	.866
NNRTI exposition duration (per 10 years increase)	−2.12 (−4.04 to 0.19)	**.031**	−1.25 (−4.01 to 1.50)	.370	0.53 (−1.40 to 2.46)	.588
INSTI exposition duration (per 10 years increase)	−1.55 (−5.87 to 2.77)	.479	0.09 (−4.50 to 4.31)	.966	0.69 (−2.37 to 3.75)	.655
Nadir CD4 count (per 100 cells/mm^3^)	−0.08 (−0.68 to 0.51)	.783	0.12 (−0.54 to 0.79)	.712	0.25 (−0.21 to 0.71)	.293
Current CD4 count (per 100 cells/mm^3^)	−0.19 (−0.46 to 0.08)	.172	−0.16 (−0.48 to 0.16)	.341	−0.21 (−0.43 to 0.02)	.072
Current CD8 count (per 100 cells/mm^3^)	0.05 (−0.15 to 0.26)	.605	0.06 (−0.15 to 0.27)	.573	0.07 (−0.08 to 0.21)	.362
Detectable viral load (yes or no)§	3.15 (0.20–6.09)	**.036**	1.62 (−1.56 to 4.79)	.316	2.21 (−1.99 to 2.43)	.844

Bold numbers to identify statistical significance.

INSTI = integrase strand transfer inhibitors, NNRTI = nonnucleoside reverse transcriptase inhibitors, NRTI = nucleoside reverse transcriptase inhibitors, PI = protease inhibitors.

* Multivariable analyses included age, sex, cardiovascular risk factors if they showed an association with the outcome in the univariable model with a *P* value .1 or less and HIV related parameters including HIV infection duration, PI exposition duration, NRTI exposition duration, NNRTI exposition duration, INSTI exposition duration, nadir CD4 count, current CD4 count, current CD8 and detectable viral load.

† Multivariable analyses included age, sex, EF volume, cardiovascular risk factors if they showed an association with the outcome in the univariable model with a *P* value .1 or less and HIV related parameters including HIV infection duration, PI exposition duration, NRTI exposition duration, NNRTI exposition duration, INSTI exposition duration, nadir CD4 count, current CD4 count, current CD8 and detectable viral load.

‡ Beta is interpreted as the mean increase in epicardial fat attenuation (in HU) predicted by the model per each one unit change in the explanatory variable. A negative beta coefficient indicates a decrease.

§ Defined as viral load greater than 40 copies/mL (0: no detectable viral load, 1: detectable viral load).

### 3.4. EF density and coronary plaque burden

Univariable analysis showed no significant association between EF density and coronary calcium score, total plaque volume, subtypes of plaque volume and low-attenuation plaque volume (all *P* ˃ .05) (Table 6). Multivariable models adjusting for CVD risk factors showed positive association between EF density and calcium score (logit component odds ratio [OR], 1.07 per 1 HU increase in EF density, *P* = .023 and OR for Poisson regression = 1.00 per 1 HU increase in EF density, *P* = .635) while no association was observed with other measures of plaque. Additional adjustment for EF volume showed that the association between EF density and calcium score remained statistically significant (logit component OR, 1.09 per 1 HU increase in EF density, *P* = .039 and Poisson component OR 1.00 per 1 HU increase in EF density, *P* = .877) (Table 6).

**Table 6:** Association of epicardial fat density with coronary plaque burden in all participants N = 260 (177 HIV+, 83 HIV−).

	Unadjusted analysis		Adjusted analysis*		Adjusted analysis†	
	Estimate (95% CI)	*P* value	Estimate (95% CI)	*P* value	Estimate (95% CI)	*P* value
Coronary artery calcium score‡	L 1.02 (0.97–1.07)	.390	L 1.07 (1.00–1.13)	**.023**	L 1.09 (1.00–1.18)	**.039**
	P 0.99 (0.98–1.00)	.208	P 1.00 (0.98–1.01)	.635	P 1.00 (0.98–1.02)	.877
Total plaque volume (cm^3^)§	L 1.00 (0.95–1.05)	.948	L 1.04 (0.98–1.10)	.202	L 1.06 (0.95–1.15)	.201
	P 0.99 (0.98–1.01)	.358	P 1.00 (0.98–1.01)	.706	P 1.01 (0.99–1.03)	.618
Calcified plaque volume (cm^3^)∥	L 1.00 (0.96–1.06)	.842	L 1.03 (0.98–1.09)	.254	L 1.05 (0.97–1.14)	.242
	P 0.99 (0.98–1.01)	.297	P 0.99 (0.98–1.01)	.573	P 1.00 (0.97–1.03)	.899
Non calcified plaque volume (cm^3^)¶	L 0.96 (0.90–1.03)	.252	L 0.98 (0.91–1.05)	.492	L 1.01 (0.91–1.12)	.842
	P 1.01 (0.98–1.03)	.668	P 1.01 (0.98–1.04)	.661	P 1.02 (0.97–1.07)	.438
Mixed plaque volume (cm^3^)#	L 1.00 (0.95–1.05)	.990	L 1.05 (0.99–1.11)	.096	L 1.07 (0.98–1.16)	.121
	P 1.00 (0.98–1.01)	.604	P 1.00 (0.98–1.02)	.942	P 1.01 (0.98–1.03)	.663
Low attenuation plaque volume (cm^3^)**	L 0.99 (0.94–1.05)	.820	L 1.03 (0.97–1.10)	.287	L 1.05 (0.96–1.15)	.253
	P 0.99 (0.98–1.01)	.249	P 1.00 (0.98–1.01)	.742	P 1.01 (0.99–1.03)	.389

Bold numbers to identify statistical significance.

CI = confidence interval, L = logistic part, P = poisson part.

* Analysis were adjusted for age, sex and cardiovascular risk factors if they showed an association with the outcome in the univariable model with a *P* value .1 or less.

† Analysis were adjusted for age, sex and cardiovascular risk factors and EF volume they showed an association with the outcome in the univariable model with a *P* value .1 or less.

‡ Adjusted for age, sex, HIV status, high blood pressure, smoking exposure, LDL-cholesterol, and statin use.

§ Adjusted for age, sex, HIV status, smoking exposure, and statin use.

∥ Adjusted for age, sex, HIV status, smoking exposure, LDL-cholesterol, and statin use.

¶ Adjusted for age, sex, HIV status, family history of premature CVD, and statin use.

# Adjusted for age, sex, HIV status, smoking exposure, triglycerides, and stain use.

** Adjusted for age, sex, HIV status, smoking exposure, and statin use.

### 3.5. Association of EF density with metabolic and inflammatory biomarkers

Table 7 summarizes the results of the association of EF density with metabolic and inflammatory markers among 123 participants with available values (76 HIV+, 47 HIV−). Among the soluble biomarkers measured in our study, univariable regression analyses showed that only IL2Rα, IL7, leptin, peptid C, insulin, TNFα, EAN78, growth-regulated oncogene alpha, follicular stimulating hormone, luteizing hormone (LH) and PP were associated with EF density. After adjustment for CVD risk factors and EF volume, only IL2Rα, TNFα and LH remained significantly associated with EF density.

**Table 7:** Association of epicardial fat density with metabolic and inflammatory markers in participants living with HIV and controls, N = 123 (76 HIV+, 47 HIV−).

	Unadjusted analysis		Adjusted analysis*		Adjusted analysis†	
	Beta (95% CI)	*P* value	Beta (95% CI)	*P* value	Beta (95% CI)	*P* value
IL2RA‡	0. 24 (0.03–0.45)	**.024**	0.28 (0.08–0.48)	**.006**	0.19 (0.05–0.34)	**.010**
IL7	−0.12 (−0.22 to 0.01)	**.025**	−0.08 (−0.19 to 0.02)	.099	−0.07 (−0.15 to 0.01)	.075
Leptine‡	−0.02 (−0.03 to 0.01)	**<.001**	−0.02 (−0.03 to 0.01)	**.004**	−0.01 (−0.02 to 0.00)	.078
Peptid C‡	−0.07 (−0.12 to 0.03)	**.002**	−0.04 (−0.09 to 0.01)	.080	−0.01 (−0.05 to 0.02)	.522
Insuline	−0.04 (−0.07 to 0.01)	**.003**	−0.02 (−0.05 to 0.01)	.146	−0.01 (−0.03 to 0.01)	.395
TNF alpha	1.11 (0.12–2.10)	**.029**	1.48 (0.52–2.44)	**.002**	0.92 (0.23–1.63)	**.010**
ENA78‡	−0.03 (−0.07 to 0.001)	**.045**	−0.02 (−0.06 to 0.01)	.162	−0.01 (−0.04 to 0.01)	.207
GRO alpha‡	−0.19 (−0.34 to 0.03)	**.016**	−0.16 (−0.31 to 0.00)	**.045**	−0.08 (−0.20 to 0.03)	.165
FSH‡	0.02 (0.00–0.04)	**.049**	0.02 (0.00–0.05)	**.019**	0.01 (0.00–0.03)	.055
LH‡	0.04 (0.00–0.08)	**.038**	0.04 (0.00–0.08)	**.038**	0.03 (0.00–0.06)	**.029**
PP‡	0.35 (0.05–0.65)	**.021**	0.27 (−0.03 to 0.59)	.075	0.15 (−0.08 to 0.38)	.197

Bold numbers to identify statistical significance.

ENA = epithelial-derived neutrophil-activating, FSH = follicular stimulating hormone, GRO = Growth-regulated oncogene, IL = interleukine, LH = luteizing hormone, TNF = tumor necrosis factor.

* Analysis were adjusted for age, HIV status and CAD risk factors (diabetes, high blood pressure, family history of CVD, smoking exposure in pack/year, LDL-cholesterol, HDL-cholesterol, triglyceride, BMI and statin use) if they showed an association with the outcome in the univariable model with a *P* value .1 or less.

† Analysis were adjusted for age, HIV status, EF volume and CAD risk factors (diabetes, high blood pressure, family history of CVD, smoking exposure in pack/year, LDL-cholesterol, HDL-cholesterol, triglyceride, BMI and statin use) if they showed an association with the outcome in the univariable model with a *P* value .1 or less.

‡ Per 100 unit increase.

## 4. Discussion

### 4.1. Main findings of the study

In the current cross-sectional study, we showed that EF density did not differ by HIV status. EF density was positively associated with male sex, diabetes, and coronary calcium score, independent of cardiovascular risk factors and EF volume, but no association was found with HIV specific factors. Finally, we found that EF density was independently associated with some metabolic and inflammatory markers, mostly IL2Rα, TNFα, and LH.

### 4.2. EF density

PLHIV have a higher risk to develop CAD with subsequent complications, as shown in large cohort studies.^[3–5]^ Mechanisms underlying this increased risk are not yet fully understood but several factors such as ART, abnormal fat deposition, inflammation or immune activation may play a role.

HIV infection and use of ART can lead to alterations in fat distribution.^[29,30]^ We and others have previously showed that quantity of EF was increased in PLHIV compared to uninfected controls.^[10,11,31]^ Studies in the general population as well as in PLHIV have demonstrated that greater quantity of EF was associated with a greater coronary atherosclerotic plaque burden.^[11,32,33]^ EF secretes bioactive cytokines that are known to promote atherosclerosis.^[8,34]^

Recently, assessment of EF quality has gained interest. However, only a few studies reported results specific to the HIV population.

### 4.3. EF density and HIV infection

Unexpectedly, PLHIV in our cohort did not have a significantly different EF density compared to healthy controls, despite having a higher volume of EF. As in our study, Buggey et al,^[35]^ did not find a significant difference between HIV-positive and HIV-negative participants when comparing EF density measured in the left atrium roof and peri-right coronary artery. In another study evaluating density of abdominal visceral fat, fat density did not differ by HIV status.^[36]^ EF inflammation and fibrosis may had alleviated the expected decrease in fat density and therefore explain the absence of difference in fat density between HIV-positive and HIV-negative participants despite the difference in EF volume.

In our study, we observed an inverse correlation between EF density and NNRTI exposure duration and positive association with detectable viral load in univariable analyses. However, these associations did not persist after adjustment for CVD risk factors and EF volume. In their study, Longenecker et al^[37]^ found that baseline EF density and changes over time were inversely correlated with ART and duration of protease inhibitor exposure. Similarly, studies evaluating abdominal visceral fat demonstrated that exposure to ART was associated with a lower density of fat.^[16,38,39]^ We and others have demonstrated that a longer exposure to ART and to some specific ART classes were associated with an increased EF volume.^[10,11]^

### 4.4. EF density and coronary plaque burden

To our knowledge, our study is the first to evaluate the association of EF density with coronary artery plaque burden in PLHIV. We found that EF density was positively associated with coronary calcium score in multivariable models adjusting for CVD risk factors and EF volume.

Our results are consistent with findings in the general population. In their studies, Pracon et al^[40]^ and Liu et al^[18]^ showed that EF density was positively correlated to coronary calcium score. Other studies demonstrated a positive association of EF density with coronary atherosclerosis severity as well as with myocardial infarction.^[13,18,41]^ Higher EF radiodensity may reflect fat inflammation and fibrosis and represent a form of unfavorable metabolic activity which may directly affect coronary atherosclerosis. The association of EF density with coronary plaque burden was independent of EF volume suggesting that different mechanisms may account for clinical effect. Of note, some groups have reported opposite association between EF density and coronary plaque and showed that decreased density was associated with impaired CAD profile.^[42–44]^ This may be explained by the inclusion of patients with different degrees of inflammation and fibrosis in EF.

Future mechanistic studies will need to be carefully designed to unravel the complex relationship between fat quality and CAD.

### 4.5. EF density and metabolic and inflammatory biomarkers

Fat abnormalities in PLHIV have been associated with metabolic dysregulation, inflammation, and immune activation. In our study, we evaluated a selected panel of metabolic and inflammatory markers and showed that only IL2Rα, TNFα and LH were positively associated with EF density independent of CVD risk factors and EF volume. IL2 receptor subunit alpha plays an important role in the proliferation and differentiation of T cells.^[45,46]^ High levels of IL2Rα have been associated with multiple inflammatory diseases such as rheumatoid arthritis,^[47]^ as well as with CAD.^[48]^ TNFα represents one of the most potent pro-inflammatory cytokines. Studies have demonstrated that TNF-α has roles in the development of atherosclerosis.^[49,50]^ Finally, recent findings demonstrated that adipose tissue regulates the LH secretion via leptin production.^[51]^ Elevated levels of LH are associated with abnormalities predisposing to CVD risk such as insulin resistance, dyslipidemia, and systemic inflammation.^[52,53]^

Our findings support the hypothesis that an abnormal EF quality may be linked to subclinical atherosclerosis through its underlying source of inflammatory mediators potentially favoring atherosclerosis. Consequently, CT measurements of quality of EF might add valuable information in the cardio-vascular risk assessment of HIV individuals.

Only few studies evaluated the correlation of fat density with inflammatory markers in PLHIV and found results comparable to ours. Longenecker et al,^[37]^ and Chen et al,^[54]^ showed that density of EF was negatively correlated to hs-CRP, IL6 and positively correlated T-cell activation. Similarly, other studies evaluating abdominal visceral fat density showed that it was negatively correlated to hs-CRP, IL6 and leptin level and positively correlated to adiponectin.^[36,38]^

### 4.6. Strengths and limitations

Our study has many strengths including its robust design, the use of CT that allowed us to characterize fat radiodensity in addition to volume and coronary plaque burden without additional exposition to radiation. We evaluated EF density in a population of individuals living with HIV and healthy controls, male and female and demonstrated for the first time that EF density was positively correlated to calcium coronary score in a population that includes PLHIV and to metabolic and inflammatory markers independent of EF volume. However, our study did not have sufficient power to assess whether this link was specific to PLHIV.

There are several limitations of our study: First, there may be significant regional variation of adipose tissue characteristics, particularly around atherosclerotic plaques. Future studies in the HIV population should consider more granular characterization of radiodensity by location. As we have previously reported, our study was conducted in participants who were predominantly male. It remains unknown whether these findings can be generalized to women. Although this does not bias our results, it limits their generalizability. Finally, this was a cross-sectional analysis which does not allow for determination of causality. Additionally, as a small group of participants had calcified, noncalcified or mixed plaques, the analyses on plaque sub-types may have lacked statistical power. This was also true for antiretroviral therapy, where a small group of participants were in each sub-class of ART. We performed a fair number of statistical tests, especially for the association of EF density and metabolic and inflammatory markers and we chose not to adjust p values for multiple comparisons which may have increase the likelihood of obtaining false positive results. But this was a hypothesis-generating study and further studies are needed to confirm our findings.

As PLHIV live longer, understanding the relationship between HIV, ART, and contributors to CAD risk is important. Our study shows that an increase in EF density is associated with a higher coronary calcium score in a population including PLHIV. An increase in EF density is also associated to metabolic and inflammatory dysfunction. Future measurement of EF density may provide additional insight into adipose tissue function beyond measurement of quantity alone and may have implications for assessment of long-term cardiovascular risk.

## Acknowledgements

We would like to thank all the staff and participants for their contribution in the actual study including: Stéphanie Matte, Annie Chamberland and Nathalie Bellavance.

## Author contributions

**Conceptualization:** Manel Sadouni, Cécile Tremblay, Carl Chartrand-Lefebvre, Madeleine Durand.

**Data curation:** Marie Duquet-Armand, Mohamed Ghaiss Alkeddeh, Etienne Larouche-Anctil, Jean-Guy Baril, Benoit Trottier, Carl Chartrand-Lefebvre, Madeleine Durand.

**Formal analysis:** Manel Sadouni.

**Funding acquisition:** Cécile Tremblay, Carl Chartrand-Lefebvre, Madeleine Durand.

**Investigation:** Madeleine Durand.

**Methodology:** Manel Sadouni, Cécile Tremblay, Carl Chartrand-Lefebvre, Madeleine Durand.

**Supervision:** Madeleine Durand.

**Writing – original draft:** Manel Sadouni.

**Writing – review & editing:** Manel Sadouni, Cécile Tremblay, Jean-Guy Baril, Carl Chartrand-Lefebvre, Madeleine Durand.
